# Clinical, Radiographic, and Histomorphometric Evaluation of a Vertical Ridge Augmentation Procedure Using a Titanium-Reinforced Microporous Expanded Polytetrafluoroethylene Membrane: A Prospective Case Series with 1-Year Follow-Up

**DOI:** 10.3390/ma14143828

**Published:** 2021-07-08

**Authors:** Jung-Gu Ji, Jung-A Yu, Seong-Ho Choi, Dong-Woon Lee

**Affiliations:** 1 Department of Periodontology, Dental Hospital, Veterans Health Service Medical Center, Seoul 05368, Korea; jjg0414@naver.com (J.-G.J.); terrace71@hanmail.net (J.-A.Y.); 2 Department of Periodontology, College of Dentistry and Research Institute for Periodontal Regeneration, Yonsei University, Seoul 03722, Korea; SHCHOI726@yuhs.ac

**Keywords:** alveolar ridge augmentation, bone regeneration, dental implantation

## Abstract

Vertical ridge augmentation for long-term implant stability is difficult in severely resorbed areas. We examined the clinical, radiological, and histological outcomes of guided-bone regeneration using novel titanium-reinforced microporous expanded polytetrafluoroethylene (MP-ePTFE) membranes. Eighteen patients who underwent implant placement using a staged approach were enrolled (period: 2018–2019). Vertical ridge augmentation was performed in areas with vertical bone defects ≥4 mm. Twenty-six implant fixtures were placed in 14 patients. At implant placement six fixtures had relatively low stability. On cone-beam computed tomography, the average vertical changes were 4.2 ± 1.9 (buccal), 5.9 ± 2.7 (central), and 4.4 ± 2.8 mm (lingual) at six months after vertical ridge augmentation. Histomorphometric analyses revealed that the average proportions of new bone, residual bone substitute material, and soft tissue were 34.91 ± 11.61%, 7.16 ± 2.74%, and 57.93 ± 11.09%, respectively. Stable marginal bone levels were observed at 1-year post-loading. The residual bone graft material area was significantly lower in the exposed group (*p* = 0.003). There was no significant difference in the vertical height change in the buccal side between immediately after the augmentation procedure and the implant placement reentry time (*p* = 0.371). However, all implants functioned well regardless of the exposure during the observation period. Thus, vertical ridge augmentation around implants using titanium-reinforced MP-ePTFE membranes can be successful.

## 1. Introduction

Over the last 30 years, guided bone regeneration (GBR) has achieved predictable results in patients with missing teeth [1]. Several methods of augmentation using GBR for implant placement have been investigated in patients with severely absorbed alveolar conditions [2]. Although horizontal bone defects have achieved relatively predictable results with GBR [3,4], vertical ridge augmentation remains challenging because of the complexity of techniques and the complication risk.

Vertical ridge augmentation is needed to ensure the stability of blood clots and graft materials during new bone formation [5]. Moreover, tissue engineering approaches, such as mesenchymal stem cells and bioactive scaffolds in the regenerative healing of alveolar bone area, are also actively performed [6,7,8]. We should be considered as the recent development of tissue engineering showed effective outcomes on bone regeneration [9]. However, the use of resorbable membranes for vertical bone augmentation had limited success [10]. Therefore, titanium mesh and titanium-reinforced nonresorbable membranes have been introduced [11]. The use of a titanium mesh for space maintenance during vertical ridge augmentation has demonstrated promising results for bone quantity; however, this approach also has some limitations, including the risk of membrane exposure because of sharp edges, stiffness, and difficulties during removal [12]. In addition, expanded polytetrafluoroethylene (ePTFE) membranes can be contaminated by bacteria because of relatively large pore sizes, are difficult to remove because of soft-tissue ingrowth excess, can lead to serious complications from membrane exposure [10], and are difficult to obtain.

Therefore, dense PTFE (dPTFE) membranes with smaller pore sizes (<0.3 µm) have been developed with variable results. The limitations of ePTFE membranes, including the lack of occlusivity and difficulty during removal, have been improved using dPTFE membranes [13]. Moreover, unlike ePTFE membranes, primary closure may not be necessary with dPTFE membranes [14]. However, there is a tendency for early sloughing or exposure when a dPTFE membrane is used during bone augmentation because of poor-tissue attachment to its smooth surface and stiffness.

Recently, a novel titanium-reinforced microporous ePTFE membrane (MP-ePTFE) with a reduced pore size (<0.3 µm) created by ePTFE membrane processing was introduced. However, the research related to the use of this membrane for vertical bone augmentation is limited. Therefore, in this prospective case series, we investigated the effectiveness of this membrane using clinical, radiological, and histomorphometry evaluations. Additionally, we aimed to evaluate factors according to the clinical outcomes after using a titanium-reinforced MP-ePTFE membrane for vertical ridge augmentation before dental implant placement.

## 2. Materials and Methods

### 2.1. Study Design

This study was conducted in patients who required implant restoration but lacked vertical bone volume in the area. Between July 2018 and April 2019, 18 patients who underwent implant placement via a staged approach were enrolled.

**Figure 1:**
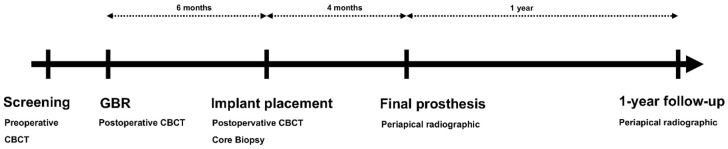
Study design. CBCT, cone-beam computed tomography; GBR, guided bone regeneration.

Patients with a suitable oral hygiene for oral surgery, including implant placement, and those needed implant placement but lacked sufficient bone quantity, with a vertical bone defect ≥4 mm on radiographic evaluations, were enrolled. The exclusion criteria were as follows: (i) history of a systemic disease that could affect oral surgery, (ii) prescription of oral or injected bisphosphonates, or (iii) current smoking (≥10 cigarettes/day). Even if the inclusion criteria were satisfied, patients with vertical bone defects <4 mm after flap elevation, those who would not sign the consent form, and those who failed to attend follow-up appointments were also excluded from the study (Figure 1)

### 2.2. Surgical Procedures

Vertical and horizontal incisions were performed under local anesthesia. The periodontal flap was reflected, and the sizes and shapes of bone defects were identified. Vertical ridge augmentation was performed in areas with vertical bone defects ≥4 mm, as measured with a probe (Williams Probe, Hu-Friedy, Chicago, IL, USA). A releasing incision was performed for the primary closure. Then, a titanium-reinforced MP-ePTFE membrane (OpenTex^®^-TR, Purgo, Seoul, Korea) was trimmed and bent to cover at least 2–3 mm beyond the defect and at 3 mm from the adjacent teeth, with application to the palatal or lingual areas with screws (Autoscrew, Jeil, Seoul, Korea) or bone tacks (truFIX, ACE Surgical Supply Co., Inc., Brockton, MA, USA). A combination of allogenic (ICB Cortical^®^, Rocky Mountain Tissue Bank, Aurora, CO, USA) and xenogenic (The Graft, Purgo, Seoul, Korea) bone in a 1:1 or 2:1 ratio was grafted in each defect. The prepared membrane was covered with a bone graft material on the buccal side, and additional screws or bone tacks were added to fix it. Then, a tension-free flap was created and sutured with nylon (5–0 blue nylon; AILEE, Busan, Korea).

**Figure 2:**
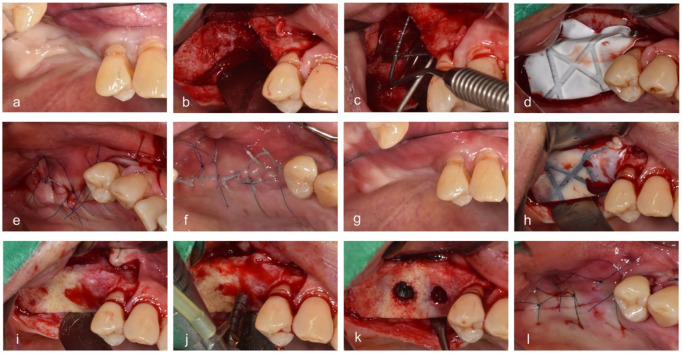
Surgical procedures. (**a**) Before vertical ridge augmentation, (**b**) flap reflection for augmentation, (**c**) measuring the defect, (**d**) grafting bone material and membrane, (**e**) suturing, (**f**) healing state when visiting for removal of stitches, (**g**) before reentry, (**h**) flap reflection for implant placement, (**i**) removing the membrane, (**j**) drilling, (**k**) implant placement, and (**l**) suturing.

Cone-beam computed tomography (CBCT) imaging (voxel size, 0.30 mm; exposure time, 8.9 Â s; 120 kVP, 18.54 mAs) was conducted immediately after surgery using a KaVo 3D eXam instrument (Imaging Sciences International LLC, Hatifield, PN, USA). Antibiotics and analgesics were prescribed for 1 week, and patients were instructed to rinse with chlorhexidine (Hexamedine; Bukwang, Seoul, Korea). After 14 days, the suture materials were removed, and clinical evaluations were conducted. The membrane was removed 6 months later at the time of implant placement, at which time another CBCT examination was performed. A biopsy with a 2.7-mm inner diameter was collected in the long-axis direction using a trephine bur (Trephine Bur kit Xit, Dentium, Seoul, Korea) at the implant placement site. Implants with appropriate diameters were placed for prosthetic treatment (Figure 2).

All surgeries were performed by one expert (L.D.W.). If an unexpected exposure occurred during the healing period, the membrane was removed or maintained depending on the clinical signs, including fluctuation, pus discharge, and movement of membrane, among others [15,16,17].

### 2.3. Clinical Analysis

#### 2.3.1. Exposure

**Figure 3:**
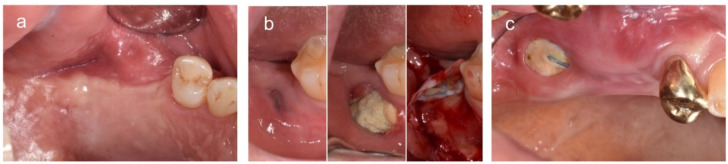
Representative cases. (**a**) No exposure, (**b**) exposure with membrane removal during the healing period, and (**c**) exposure without membrane removal until implant placement.

Exposure of a membrane and its timing were evaluated. When a membrane was exposed, it was classified as an exposure with membrane removal during the healing period or an exposure without membrane removal until implant placement. In addition, the location and size of the exposure were measured and evaluated (Figure 3).

#### 2.3.2. Primary Stability at the Time of Implant Placement

The initial stability of an implant was measured by the torque value (N/cm), which appeared on the screen of a surgical motor with torque control (Intrasurg500, Kavo, Biberach, Germany) during implant insertion. The stability was categorized as >30 or <30 N/cm.

#### 2.3.3. Additional Bone Grafts 

The requirement for an additional bone graft at the time of implant placement was also evaluated.

### 2.4. Radiographic Analysis

#### 2.4.1. CBCT

Radiographic measurements and analyses were performed by J.G.J. under the supervision of a senior author (D.W.L.). CBCT images obtained at baseline (T0), immediately after surgery (T1), and at six months after surgery (T2) were used for these analyses. These CBCT scans were superimposed based on specific reference points (e.g., the cranial base, external and internal oblique ridges, and inferior border of mandible), and best-matched cuts were obtained with additional corrections [18].

**Figure 4:**
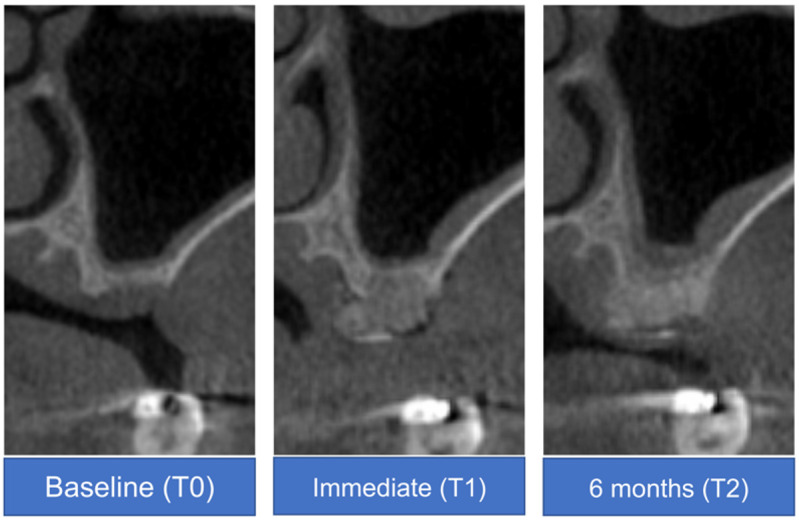
Cone-beam computed tomography analysis of a representative case at baseline (T0), immediately after surgery (T1), and at 6 months after surgery (T2).

A vertical reference line was set at the center of the alveolar bone, parallel to the long axis of the adjacent tooth. Two vertical lines parallel to this reference line were formed on the buccal and lingual sides. Vertical height changes in the buccal, mid, and lingual vertical reference lines (VHB, VHM, and VHL, respectively) were measured and analyzed [19] (Figure 4).

#### 2.4.2. Periapical Radiography

Periapical radiographs were obtained immediately after surgery and at 1 year after completion of the final prosthesis to compare the heights of the mesial and distal marginal bones using a film holder (XCP-DS FIT, Dentsply, Waltham, MA, USA) with the long-cone paralleling technique. Distances on each side were calculated using a digital caliper via a radiographic viewer (mViewer, Marotech, Seoul, Korea) [20]. Marginal bone loss was measured to evaluate marginal bone stability.

### 2.5. Histological Processing and Histomorphometry Analysis

Bone cores obtained during implant placement were fixed in 10% buffered neutral formalin (Sigma-Aldrich, St. Louis, MO, USA) for 14 days. Then, the bone cores were decalcified in 5% formic acid and embedded in paraffin. Serial perpendicular sections (5-μm thickness) were cut along the center of each specimen, and the central-most sections were stained with hematoxylin and eosin. Histomorphometric analysis was performed using image analysis software (Photoshop CS6, Adobe, CA, USA). The percentages of newly formed bone, residual bone graft material, soft tissue, and background were measured.

### 2.6. Statistical Analysis

Data are presented as means ± standard deviations for continuous variables or as numbers with percentages for categorical variables. Even if there were two or three sites per patient, only one biopsy was performed during drilling for preparation of implant placement. Normality of variables was tested with the Shapiro-Wilk test. The enrolled areas (one area per patient) were analyzed to compare the baseline characteristics and outcomes of exposed versus nonexposed groups using the two-sample t-test or Fisher’s exact test. Especially, to evaluate the change of vertical height according to the group and time, we conducted a generalized least square linear model analysis. The statistical significance level was set at *p* < 0.05. All analyses were conducted using R software 4.0.1 (R Foundation for Statistical Computing, Vienna, Austria).

## 3. Results

### 3.1. Demographic Information

**Figure 5:**
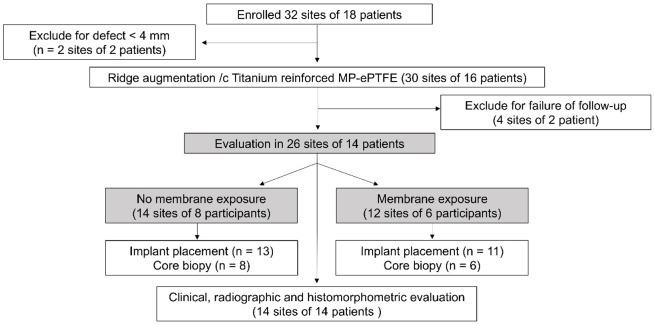
Study flow. MP-ePTFE, microporous expanded polytetrafluoroethylene.

In total, 18 eligible patients consented to participate in this study; however, two patients were excluded because the heights of their deepest areas were <4 mm after flap elevation. Sixteen patients subsequently underwent vertical bone augmentation with a titanium-reinforced MP-ePTFE membrane; however, two of these patients were not followed up and, therefore, they were excluded (Figure 5, Table 1).

In cases with multiple teeth, CBCT analysis was performed on the site showing the largest vertical defect. In total, 26 sites in these 14 patients (sex, nine men and five women; mean age, 67 ± 9.3 years) were evaluated. In all cases, tooth extraction was the origin of the periodontal disease.

### 3.2. Clinical Analysis

Of the 26 sites, six had membrane exposure. Three sites underwent membrane removal during the healing period due to failure of fixation (exfoliation of screw) or mild suppuration. The other three sites were exposed without membrane removal until implant placement (Figure 4). Regarding the exposure location, one site was exposed around the crestal area, and five sites were exposed beyond the mucogingival junction. In total, 24 implant fixtures were planned at the 26 sites. Of these 24 fixtures, 18 and six fixtures showed good (≥30 N/cm) and relatively low initial stability (<30 N/cm), respectively. At implant placement, only one patient required an additional bone graft. All 24 implants were successful and functioned well during the 1-year follow-up period.

### 3.3. Radiographic Analysis

#### 3.3.1. CBCT

At baseline (T0), the average vertical heights were 9.8 ± 8.8, 9.3 ± 8.0, and 10.6 ± 8.2 mm at the VHB, VHM, and VHL, respectively. Immediately after vertical ridge augmentation (T1), the average vertical heights were 15.2 ± 8.8, 16.0 ± 9.0 mm, and 16.0 ± 8.6 mm at the VHB, VHM, and VHL, respectively. After six months (T2), the average vertical heights were 14.0 ± 8.0, 15.1 ± 8.6, and 15.0 ± 8.0 mm at the VHB, VHM, and VHL, respectively. The average T2-T0 changes were 4.2 ± 1.9, 5.9 ± 2.7, and 4.4 ± 2.8 mm at the VHB, VHM, and VHL, respectively.

#### 3.3.2. Periapical Radiography

Between the start of function and at the 1-year follow-up period, the differences in the marginal bone levels (the distance from the implant-abutment connection to the top of the crestal bone) were 0.16 ± 0.05 and 0.15 ± 0.04 mm in the mesial and distal areas, respectively.

### 3.4. Histomorphometric Analysis

Biopsies were obtained from 14 patients, with evidence of significant bone marrow tissue formation, new bone around residual bone, and bone graft material. Little inflammation-related tissue was observed.

The histomorphometric values for new bone, bone material, and soft tissue were 34.91 ± 11.61%, 7.16 ± 2.74%, and 57.93% ± 11.09%, respectively.

**Figure 6:**
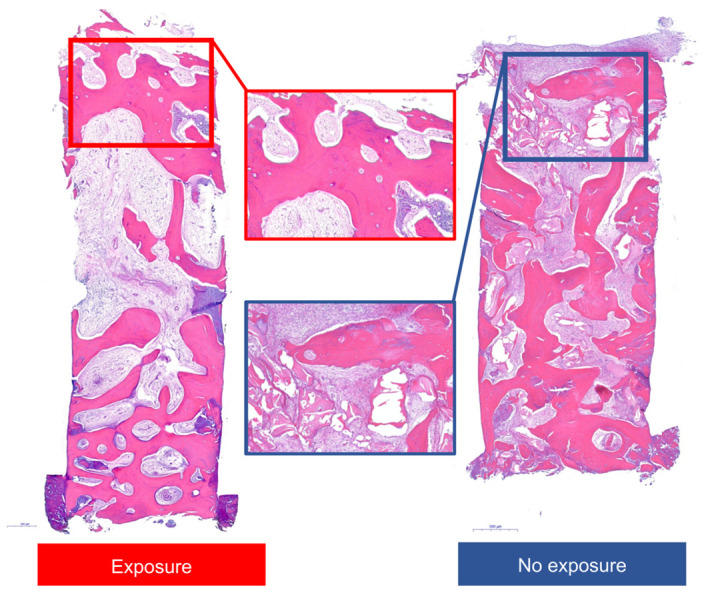
Histologic views of representative specimens with or without exposure. Images represent entire and high-magnification views.

Epithelialization was relatively more reduced in cases with membrane exposure compared to those without exposure; however, there was no evidence of less new bone formation in either group (Figure 6).

### 3.5. Statistical Analysis

There were no significant differences identified in baseline characteristics (e.g., sex, age, sites, single/multiple, smoking, and re-entry period) for analysis of factors affecting exposure (Table 2). There were also no significant differences in the outcomes of vertical augmentation (i.e., primary stability, marginal bone level, and histomorphometric analysis) between the exposed and nonexposed groups. However, there was a significantly lower area of residual bone graft material in the exposed group (*p* = 0.003).

**Figure 7:**
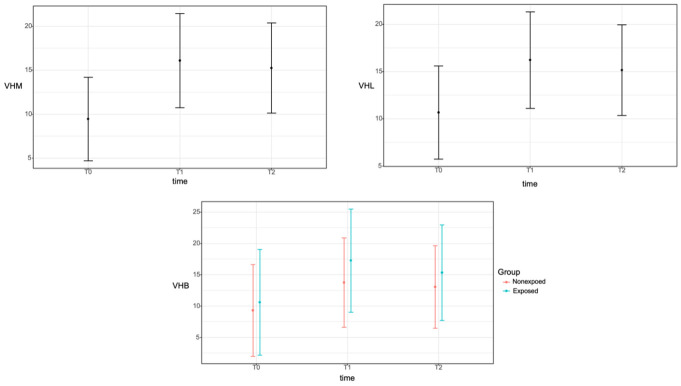
Correlation between the time (baseline, T0; immediately after surgery, T1; and at 6 months after surgery, T2) and the vertical height change in the VHB, VHM, and VHL reference lines using cone-beam computed tomography. The mean values are expressed as closed circles, and confidence intervals are represented by error bars. For the VHB, group, time, and interaction variables were included in the model because the interaction was significant. There was no significant difference in the VHB at T1 and T2 time points in the non-exposed group (*p* = 0.371), but there was a significant difference between all other time points in the non-exposed and exposed groups (*p* < 0.05). VHB, buccal reference line; VHM, mid reference line; VHL, lingual reference line.

The generalized least squares model was utilized to examine the correlation of time in the VHM and VHL. In the case of VHM and VHL, the interaction and group variables were not significant and were removed from the model. There were significant differences between all-time points regardless of exposure (*p* < 0.05) (Table 3). In the VHB, group, time, and interaction variables were included in the model because the interaction was significant. There was no significant difference in the VHB at T1 and T2 time points in the non-exposed group (*p* = 0.371), but there was a significant difference between all other time points in the non-exposed and exposed groups (*p* < 0.05) (Figure 7).

## 4. Discussion

To the best of our knowledge, this is the first study that reported the use of a titanium-reinforced MP-ePTFE membrane for vertical ridge augmentation before dental implant placement. This study demonstrated that a titanium-reinforced MP-ePTFE membrane can successfully be used for vertical ridge augmentation of severely resorbed ridges in posterior areas with or without exposure. A previous study demonstrated that exposure of resorbable membranes resulted in relatively low rates of healing, with additional fixation required for horizontally resorbed ridges [21]. In addition, vertical ridge augmentation using an ePTFE membrane, which is obtained by stretching of PTFE insulation at high temperatures, has distinct challenges. For example, the comparatively large pore size of this membrane provides an easy pathway for bacterial contamination. In addition, surgical removal of contaminated membranes can be complicated because of excessive soft tissue ingrowth [22]. Moreover, upon exposure of an ePTFE membrane, bone regeneration is reduced and soft tissue dehiscence occurs. Finally, it is currently impossible to use titanium-reinforced ePTFE membranes, which have been studied for GBR for decades.

**Figure 8:**
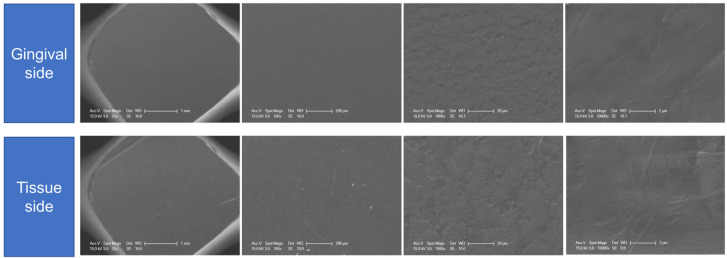
Scanning electron microscopy images of the gingival and bone sides of the microporous expanded polytetrafluoroethylene membrane evaluated in this study. Images show high-magnification views on each side.

Dense, nonporous PTFE membranes, or dPTFEs, with smaller pore sizes (<0.3 µm) were developed to overcome these limitations of ePTFE membranes; these membranes prevent the accumulation of microorganisms and facilitate easy removal of the membrane material after tissue regeneration. However, dPTFE membranes are stiffer, and the smoothness of their surface makes attachments with cells and tissues difficult, often resulting in early flap sloughing and exposure. In addition, the stiffness of these membranes can increase their susceptibility to premature exposure because they tend to revert to their original shapes after adapting to cover bone defects [23]. Thus, the pore sizes of non-resorbable membranes have been adjusted with the development of expanded membranes with smaller pore sizes (<0.3 µm), which can be separated during the initial stages of ePTFE creation (Figure 8).

Failure of GBR using nonresorbable membranes is mainly associated with membrane exposure, which can result in infection, contamination, and impaired bone augmentation [24]. Some of these complications, including abscess formation with purulent exudates, can lead to a complete GBR failure [15]. It is known that ePTFE membranes have premature exposure rates of 30–40%, accompanied by suppuration and a significant risk of infection [10]. In a previous study, dPTFE membranes were shown to have a premature exposure rate of 25.7% [25]. In this case series, 42.8% (six out of 14 cases) of patients demonstrated premature exposure, which represented no less exposure than expected. Various factors, including the amount of keratinized gingiva, flap thickness, tension, type and size of the bone defect, membrane type, and the surgeon’s experience levels can affect the exposure risk [23,26]. In this study, the patients’ older gingival phenotype may have contributed to this rate. Additionally, vertical augmentation >4 mm was more challenging in some patients who had experienced severe resorption because of periodontitis.

A previous study reported that infected sites showed insufficient ridges for implant placement after three–four months of GBR [27]. In the present study, however, three exposed sites were maintained without removal, and placement of implants with appropriate diameters and lengths was possible without additional bone grafting. Three other sites required removal of membranes because of exfoliation of screws or mild suppuration. Only one of these sites required a minor bone graft for placement of an implant with the proper diameter.

On CBCT evaluation, the generalized method of least square showed that there was no significant difference in the VHB at T1 and T2 time points in the non-exposed group (*p* = 0.371). Therefore, the buccal bone graft site in the unexposed group was well maintained during the six-month healing period immediately after the vertical augmentation procedure. However, there was a significant difference between all other time points in the nonexposed and exposed groups (*p* < 0.05) The sites with exposure did not demonstrate lower vertical gains or less new bone formation, in contrary to those without exposure. In a meta-analysis of previous studies, the mean vertical bone gain was 4.18 mm during GBR for vertical ridge augmentation [5]. In previous studies of ePTFE or dPTFE membranes for vertical ridge augmentation, the mean vertical gains in defects were 4.91 ± 1.78 and 5.49 ± 1.58 mm with ePTFE and dPTFE membranes, respectively [9]. The vertical bone gains in this study (4.2 ± 1.9, 5.9 ± 2.7, and 4.4 ± 2.8 mm for VHB, VHM, and VHL, respectively) may, therefore, reflect a more favorable result than those observed in these previous studies. Loading applied after the delivery of final restoration can effect on the bone regeneration results with the process of remodeling [28]. Despite the limitations in two-dimensional imaging, periapical radiography has been used to evaluate marginal bone stability after functioning has begun [29]. Although various criteria for success have been used, the marginal bone differences shown in this study are acceptable based on the results of other studies [30,31].

On histomorphometric analysis of biopsies, 34.91 ± 11.61% of new bone formation was observed. Previous studies have shown mean new bone formations of 18.28%, 32.6%, 36.47%, and 39.7% on histomorphometric analyses [32,33,34,35]. Histomorphometric analysis also showed less epithelialization when the membranes were exposed; however, adequate new bone formation was still achieved. In addition, secondary wound healing, which is required when exposure occurs, was observed without any severe complications, likely because of the membrane characteristics. Although the sample size was small, the exposed group showed smaller residual bone graft areas than did the nonexposed group, as expected. It is possible that premature exposure led to failures in fixation and stabilization of overlying tissue, resulting in an inability to stabilize bone graft materials. Uneventful soft tissue healing occurred, however, without exposure, as desired [36]. This soft tissue healing provides vascular and nutrient supplies to surgical sites, creates a protective barrier against biological and mechanical stimulation, and reduces the mobilization of graft materials [26].

The GBR outcomes cannot, however, solely be explained by the characteristics of the barrier membrane. This case series also supports the space-maintaining ability of titanium reinforcement, which might have also affected the results.

Several limitations of this study need to be addressed. This study was not a comparative study and, therefore, no control/study groups were used. Rather, it was a prospective case series study, in which 14 participants underwent procedures using approved products according to indications. Additional classification and statistics were performed to analyze factors according to the clinical results (exposure vs. nonexposure). Thus, there was a little consideration regarding the sample size, which was a significant limitation. Additional long-term retrospective studies will be needed to support our findings. This study was conducted mainly in the posterior area and not in the anterior zone. In addition, patients were relatively older in age, and there was an imbalance in the male-to-female ratio. Moreover, membrane removal was empirically dependent.

In patients with exposures >3 mm who develop abscesses, the infectious materials and inflammatory tissues must be removed immediately to avoid interference with the regenerative process [15,16]. For non-resorbable membranes, delayed membrane removal can lead to premature soft tissue complications because of an increased blood vessel supply requirement to the overlying flap. In addition, bacteria can penetrate the exposed membrane within four weeks after surgery [17]. Proper removal of the membrane can yield successful clinical results and prevent acute infections. However, the success of these steps is based on clinical experience, which can have a subjective impact on outcomes. Although some results cannot be quantified, the clinical significance of this pilot study conducted in 14 patients should be confirmed in a larger controlled, randomized study over a longer-term follow-up period.

## 5. Conclusions

There was a significantly lower area of residual bone graft material in the exposed group, and there was no significant difference in the vertical height change in the buccal side between immediately after augmentation procedure and the time of reentry for implant placement. However, all implants functioned well regardless of the exposure during the observation period. The results of this clinical study suggested that vertical ridge augmentation around implants using titanium-reinforced MP-ePTFE membrane can be successful. Further studies are needed to confirm our findings.

## Figures and Tables

**Table 1:** Demographic information of participants.

Study Variable	Descriptive Statistics
Sample size (patients/tooth sites)	14/26
Sex (male/female)	9/5
Age (years)	67 ± 9.3 (range, 50–79)
Arch (maxilla/mandible)	10/4
Site (nonmolar/molar)	11/15

**Table 2:** Baseline characteristics and outcomes in exposed and nonexposed groups.

Group	No Exposure	Exposure	*p*-Value
	(N = 8)	(N = 6)	
Sex			0.301
Female	4 (50.0%)	1 (16.7%)	
Male	4 (50.0%)	5 (83.3%)	
Age	63.5 ± 11.2	71.8 ± 4.1	0.084
Sites			1
Maxillary premolar	2 (25.0%)	1 (16.7%)	
Maxillary posterior	4 (50.0%)	3 (50.0%)	
Mandibular posterior	2 (25.0%)	2 (33.3%)	
Single vs. multiple			0.091
Single	1 (12.5%)	4 (66.7%)	
Multiple	7 (87.5%)	2 (33.3%)	
Smoking			1
Yes	1 (12.5%)	1 (16.7%)	
No	7 (87.5%)	5 (83.3%)	
Entry period (weeks)	180.2 ± 16.0	190.2 ± 43.4	0.613
Primary stability (N/cm)			0.473
<30	6 (75.0%)	6 (100.0%)	
≥30	2 (25.0%)	0 (0.0%)	
Change in marginal level (mm)			
Mesial	0.3 ± 0.3	0.4 ± 0.1	0.771
Distal	0.2 ± 0.2	0.3 ± 0.2	0.482
Histomorphometric (area %)			
New bone	28.6 ± 7.8	28.0 ± 4.0	0.857
Residual bone graft	8.7 ± 1.7	4.8 ± 2.3	0.003 *
Soft tissue	62.7 ± 8.4	67.2 ± 3.2	0.23

* Significant difference between groups at a *p*-value < 0.05, analyzed using the two-sample t-test or Fisher’s exact test.

**Table 3:** Generalized least square method for evaluate the vertical height according to groups and time.

Vertical Height	Variable	Beta Coefficient	Standard Error of Beta Coefficient	*p*-Value
VHB	Intercept	9.312	3.363	0.009
	Exposure	1.288	5.138	0.804
	T1	4.438	0.652	<0.001
	T2	3.750	0.713	<0.001
	Exposure × T1	2.229	0.996	0.032
	Exposure × T2	0.983	1.089	0.373
VHM *	Intercept	9.312	3.363	0.009
	T1	4.438	0.652	<0.001
	T2	3.750	0.713	<0.001
VHL *	Intercept	9.312	3.363	0.009
	T1	4.438	0.652	<0.001
	T2	3.750	0.713	<0.001

Cone-beam computerized tomographs were obtained at baseline (T0), immediately after surgery (T1), and at 6 months after surgery (T2). The vertical height change in the VHB, VHM, VHL, respectively. * For the VHM and VHL, group variables and interaction variables were not significant and, therefore, they were removed. VHB, buccal reference line; VHM, mid reference line; VHL, lingual reference line.

## Data Availability

The datasets generated during the current study are available from the corresponding author on reasonable request.
